# Distribution of psychological distress is stable in recent decades and follows an exponential pattern in the US population

**DOI:** 10.1038/s41598-019-47322-1

**Published:** 2019-08-19

**Authors:** Shinichiro Tomitaka, Yohei Kawasaki, Kazuki Ide, Maiko Akutagawa, Yutaka Ono, Toshi A. Furukawa

**Affiliations:** 1 Department of Mental Health, Panasonic Health Center, Tokyo, Japan; 2 Department of Health Promotion and Human Behavior, Kyoto University Graduate School of Medicine/School of Public Health, Kyoto, Japan; 3 Clinical Research Center, Chiba University Hospital, Chiba, Japan; 4 Department of Pharmacoepidemiology, Graduate School of Medicine and Public Health, Kyoto University, Kyoto, Japan; 5 Center for the Promotion of Interdisciplinary Education and Research, Kyoto University, Kyoto, Japan; 6 Department of Drug Evaluation and Informatics, School of Pharmaceutical Sciences, University of Shizuoka, Shizuoka, Japan; 7 Center for the Development of Cognitive Behavior Therapy Training, Tokyo, Japan

**Keywords:** Depression, Epidemiology

## Abstract

The prevalence of psychological distress is fairly stable in industrialised countries in recent decades, but the reasons for this stability remain unknown. To investigate the mechanisms underlying stability of psychological distress in the general population of the United States, we analysed the mathematical patterns of the distribution of psychological distress in recent decades. The present study utilised the Kessler psychological distress scale (K6) data from the 1997‒2017 United States National Health Interview Survey. We used overlap coefficients and graphical analysis to investigate the stability and mathematical patterns of the K6 distribution. Overlap coefficients and graphical analysis demonstrated that the distribution of K6 total scores was stable in the United States over the past two decades. Furthermore, the distributions of K6 total scores exhibited an exponential pattern, with the exception of the lower end of the distribution. These findings suggest that the lack of change in the prevalence of psychological distress over several decades is due to the stability of psychological distress distribution itself. Furthermore, the stability of the distribution of psychological distress over time may be linked to the exponential pattern of psychological distress distribution.

## Introduction

Psychological distress is characterised by depressive and anxiety symptoms, and is an indication of common psychiatric conditions such as depression and anxiety disorders^1^. Researchers have developed standardized scales, such as the six-item Kessler psychological scale (K6), for the assessment of psychological distress^2^. Previous clinical research has demonstrated that the K6 has a sensitivity of 0.34 and specificity of 0.96 at a cut-point $\underline{\underline{ > }}$13 to identify psychological distress associated with common mental illness^3^, although several studies have reported inconsistent findings on the psychometric properties of the K6 among different general populations^4,5^.

There has been substantial interest in understanding temporal trends in the prevalence of psychological distress in recent decades^6^. As the use of mental health services and antidepressants dramatically increased in the 1990s in industrialised countries, a decrease in the prevalence of distress or depressive symptoms during that period was expected^7^. However, the majority of studies in the US, Australia, UK, and Japan, which mostly used the K6, have reported little to no change in the prevalence of psychological distress and depression in the general population in recent decades^7–12^. Furthermore, a population-based study conducted in Taiwan using the 12-item Chinese Health Questionnaire (CHQ), which is a self-administered screening instrument widely used to identify non-psychotic mental disorders in Chinese populations^13^, showed a two-fold increase in the prevalence of common mental disorders between 1990 and 2010^14^.

Several possible explanations for the lack of improvement in the prevalence of common mental disorders in recent decades have been considered^6,8,9^. One explanation is that the increase in prevalence of psychological distress due to social distress has been masked by a concurrent increase in treatment^9^. Another explanation is that reductions in prevalence due to treatment have been masked by an increase in reporting of symptoms due to greater public awareness of common mental disorders^15^. Yet another prevalent explanation is that the increased provision of treatment is still insufficient to meet the requirements of individuals in greatest need^6^.

In the context of disease aetiology, a lack of change in the prevalence of psychological distress over several decades is noteworthy. In general, epidemiological research can demonstrate temporal trends of diseases and biological indexes. Since diseases are strongly affected by the environment which typically changes over time, the temporal trend of a disease can exhibit a distinct direction. Indeed, cardiovascular diseases, diabetes mellitus, cancer, infectious diseases, and allergic diseases have shown an apparent upward or downward trend in recent decades^16–21^. Conversely, although social distress such as natural disasters and economic crises impact upon psychological distress and depression^22–24^, most recent research has reported little to no change in the prevalence of psychological distress over recent decades, with the exception of a Taiwanese study^6,7,9–11,14^. It is thus necessary to consider why the prevalence of psychological distress is stable in recent decades.

**Figure 1:**
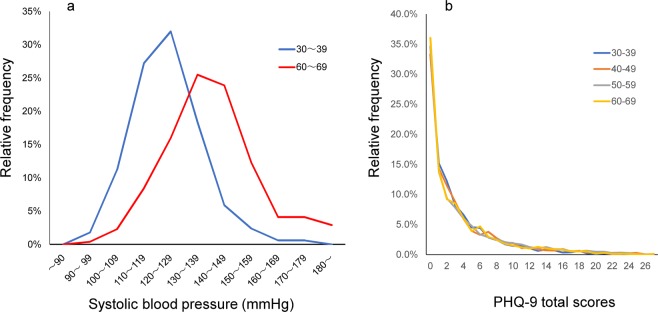
(**a**) Distributions of systolic blood pressure for Japanese men in their 30 s (n = 169) and 60 s (n = 316). The blue line represents the distribution of systolic blood pressure in Japanese men during their 30 s (mean ± S.D. = 123.2 ± 12.5 mmHg). The red line represents the distribution of systolic blood pressure in Japanese men during their 60 s (mean ± S.D. = 137.4 ± 18.4 mmHg). Data were derived from the 2014 National Health and Nutrition Examination Survey in Japan: https://www.e-stat.go.jp/stat-search/files?page=1&layout=datalist&lid=000001151595.(**b**) PHQ-9 distributions during middle adulthood. The distributions of PHQ-9 scores were similar among the middle adulthood group (30 s, 40 s, 50 s, and 60 s) in the US general population. Data were derived from the 2009‒2014 United States National Health and Nutrition Examination Survey (n = 15,847). 10.3389/fpsyt.2018.00390.

We recently reported an age-related feature of the distribution of depressive symptoms^25,26^. Biological indices, such as cardiopulmonary function, exercise ability, and brain function vary with age; in contrast, the distribution of depressive symptom scores in the general population remains considerably stable with age. For example, as shown in Fig. 1a, the distribution of blood pressure changes with age^27^. In the general population, the distribution of systolic blood pressure differs between the age of 30 and 60 years old. Conversely, the distribution of total scores in the nine-item patient health questionnaire (PHQ-9) is highly similar between 30 and 60 years of age (Fig. 1b)^25^. Generally, as the effects of age include the cumulative environmental effects on an individual, the distribution of biological indices typically vary with age. Conversely, the distribution of depressive symptoms is stable with age. These findings suggest that the distribution of depressive symptoms may be stable against environmental effects as a whole. Furthermore, the stability of the prevalence of psychological distress in recent decades may be due to the stability of the distribution itself.

Previous studies have reported mathematical features of the distributions of psychological distress and depressive symptoms^26,28,29^. These distributions follow an exponential pattern, with the exception of the lower end of the distribution in the general population^26,28–31^. Mathematically, an exponential distribution emerges when total stability and individual exchange coexist, such as income distribution and Boltzmann distribution (principle of maximum entropy)^32,33^. Thus, the stability of the prevalence of psychological distress in the general population could be linked to the exponential pattern of the distribution. However, few studies have investigated the stability of psychological distress distribution over time in the context of mathematical patterns.

For mental health-related population strategy, it is worthwhile to investigate the mathematical patterns of psychological distress distribution in the general population. Psychological distress is on a continuum in the population; high-risk individuals for common mental disorders represent the extreme end of the distribution^34^. Using an optimal cut-off point, we can estimate the prevalence rate of severe psychological distress. However, it is not necessary that this cut-off point can be used to precisely distinguish between common mental diseases and normal mental health. For example, as noted previously, Kessler *et al*. reported that K6 has a sensitivity of 0.34 at a cut-off point ≥13, indicating that almost two-thirds of individuals with common mental disorders lie below the cut-off point^2^. Rather, there is a continuous relationship between the scores on psychological distress measuring scales and the risk of common mental disorders^34–36^. Thus, for population strategy, it is important to elucidate how a scores of psychological distress, which is a risk indicator for common mental disorders, are distributed in a population.

The present study first investigated whether the distribution of total scores on the six-item Kessler psychological scale (K6) remained stable from 1997 to 2017 in the general population. Next, we examined whether the distribution of total K6 scores followed the same exponential pattern during this period. In addition, to confirm the stability of psychological distress against age, we investigated whether the distribution of K6 total scores was stable during adulthood.

## Results

### K6 total score distributions among different age groups

Table 1 shows the characteristics of the final participants and descriptive statistics for the K6 distributions according to year. The mean and median values were highly similar among all year groups. It is noteworthy that the skewness and kurtosis values for all year groups were close to 2 and 6, respectively. Mathematically, the skewness and kurtosis of any exponential distributions are 2 and 6, respectively^37^.

**Table 1:** Distribution of K6 total scores in the US general populations between 1997–2017.

Year	Number	Mean Age	Female (%)	Mean ± SD	Skewness	Kurtosis	Median	K6 score $\underline{\underline{ > }}$ 13 (%)
1997	35,625	45.9	57.1%	2.7 ± 4.1	2.1	5.0	1	4.0%
1998	31,911	46.3	56.2%	2.6 ± 4.0	2.3	6.0	1	3.5%
1999	30,304	46.5	57.2%	2.2 ± 3.7	2.5	7.1	0	2.9%
2000	31,798	46.2	56.9%	2.3 ± 3.8	2.4	6.5	0	3.1%
2001	32,397	46.1	56.6%	2.7 ± 4.0	2.1	5.2	1	3.6%
2002	30,361	46.4	56.4%	2.3 ± 3.9	2.4	6.5	0	3.4%
2003	30,154	46.5	56.6%	2.4 ± 4.0	2.3	6.0	0	3.7%
2004	30,698	47.0	55.7%	2.5 ± 3.9	2.3	5.8	1	3.6%
2005	30,895	47.4	56.3%	2.5 ± 4.0	2.3	6.1	1	3.5%
2006	23,779	46.8	55.9%	2.3 ± 3.9	2.3	6.2	0	3.3%
2007	22,982	47.2	55.7%	2.2 ± 3.7	2.5	7.3	0	2.9%
2008	21,494	47.8	56.4%	2.5 ± 4.0	2.3	5.7	1	3.5%
2009	27,442	47.6	55.9%	2.6 ± 3.9	2.2	5.7	1	3.5%
2010	26,909	47.7	55.9%	2.7 ± 4.0	2.2	5.3	1	3.8%
2011	32,735	48.0	55.2%	2.6 ± 4.0	2.2	5.4	1	3.8%
2012	34,306	48.5	55.8%	2.4 ± 3.9	2.4	6.2	0	3.5%
2013	33,280	48.6	55.3%	2.8 ± 4.2	2.1	4.8	1	4.2%
2014	35,390	49.2	55.3%	2.5 ± 3.9	2.2	5.6	1	3.5%
2015	32,256	49.9	55.2%	2.7 ± 4.0	2.1	4.8	1	3.7%
2016	31,889	50.7	54.5%	2.7 ± 4.0	2.1	5.1	1	3.9%
2017	25,767	50.9	54.7%	2.8 ± 4.0	2.1	4.8	1	3.7%
Average	30,113	47.7	55.9%	2.5 ± 3.9	2.3	5.8		3.6%

The mean psychological distress score ranged from 2.2 to 2.8 (median: 2.5). The coefficient of variation for the mean K6 score was 7.4%. Generally, a coefficient of variation with a value less than 10% indicates that the variable is considerably stable^38^. The coefficient of variation of 7.4% indicated that although the mean K6 score fluctuated from 1997 to 2017, it was stable during this period.

Linear regression analysis with mean K6 value as the dependent variable and year as the independent variable revealed that y = 0.013x + 2.386, and R^2^ = 0.1872. The slope value of 0.013 indicated a 1.3-point increase over 100 years, suggesting that the mean K6 score was stable over this time period. The low R^2^ value indicated that changes in the independent valuable were almost unrelated to changes in the dependent variable.

The prevalence of severe psychological distress ranged from 2.9% to 4.2% (mean** ± **SD: 3.6% ± 0.3%). The coefficient of variation for the mean K6 score was 9.2%, indicating that it was fairly stable during this period. Linear regression analysis with prevalence of severe psychological distress as the dependent variable and year as the independent variable revealed that y = 0.00002x + 0.03, and R^2^ = 0.1335. The low R^2^ value indicated that year had little association with the prevalence of severe psychological distress during that period.

### Overlap coefficients of the distributions among different year groups

As shown in Table 2, the overlap coefficient among different year groups ranged from 0.89 to 0.99. Of 210 combinations, only two overlap coefficients between 1999 and 2017, and between 2007 and 2017, were below 0.9. The median of the overlap coefficient was 0.96, indicating that the K6 distribution patterns were highly similar among different year groups.

**Table 2:** Overlap coefficients among different year groups.

	97	98	99	00	01	02	03	04	05	06	07	08	09	10	11	12	13	14	15	16	17
97		0.96	0.91	0.92	0.98	0.92	0.93	0.95	0.94	0.93	0.91	0.96	0.98	0.92	0.98	0.94	0.98	0.96	0.96	0.98	0.97
98			0.94	0.95	0.96	0.95	0.96	0.98	0.97	0.96	0.94	0.98	0.97	0.96	0.96	0.96	0.97	0.98	0.95	0.95	0.94
99				0.99	0.90	0.99	0.98	0.96	0.97	0.98	0.99	0.95	0.92	0.91	0.92	0.98	0.92	0.95	0.90	0.90	0.89
00					0.91	0.99	0.98	0.97	0.97	0.98	0.98	0.96	0.93	0.92	0.93	0.98	0.94	0.95	0.91	0.91	0.90
01						0.91	0.92	0.94	0.94	0.92	0.90	0.95	0.97	0.98	0.97	0.93	0.97	0.95	0.96	0.99	0.98
02							0.98	0.97	0.97	0.98	0.98	0.96	0.93	0.92	0.93	0.98	0.93	0.95	0.95	0.91	0.90
03								0.97	0.97	0.99	0.97	0.97	0.94	0.92	0.94	0.97	0.94	0.96	0.92	0.98	0.91
04									0.99	0.98	0.96	0.98	0.96	0.95	0.96	0.98	0.96	0.98	0.94	0.94	0.98
05										0.98	0.96	0.98	0.95	0.94	0.96	0.98	0.96	0.98	0.94	0.94	0.92
06											0.98	0.97	0.94	0.93	0.94	0.98	0.94	0.96	0.92	0.92	0.91
07												0.95	0.92	0.91	0.92	0.97	0.92	0.95	0.90	0.90	0.89
08													0.97	0.95	0.97	0.97	0.97	0.99	0.95	0.95	0.94
09														0.98	0.99	0.95	0.98	0.97	0.96	0.98	0.97
10															0.98	0.94	0.97	0.96	0.96	0.99	0.98
11																0.95	0.98	0.97	0.96	0.97	0.96
12																	0.95	0.97	0.92	0.93	0.92
13																		0.97	0.97	0.97	0.96
14																			0.95	0.95	0.94
15																				0.97	0.95
16																					0.98
17																					

### Graphical analysis of the distributions among different year groups

**Figure 2:**
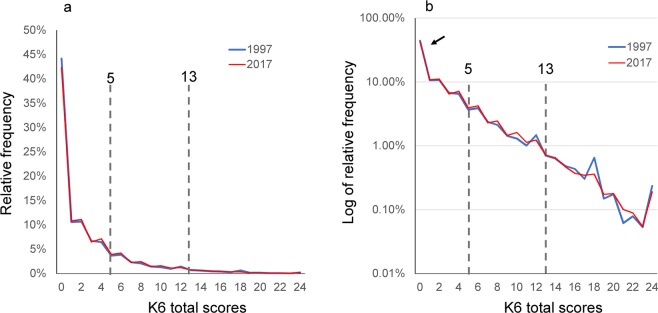
(**a**) Comparison of K6 distributions between 1997 and 2017 groups. The two graphs for 1997 and 2017 appear as a single graph as they almost completely overlapped. (**b**) On a log-normal scale, the distributions for 1997 and 2017 exhibited a linear pattern with similar gradients on a log-normal scale, indicating that K6 total scores for the two groups followed an exponential pattern with similar rate parameters on a normal scale. As indicated by arrows, at the lower end, distributions for all groups exhibited higher frequencies compared to those predicted from the exponential pattern. Auxiliary lines indicate the cut-points for moderate and severe psychological distress (a score of 5 and 13, respectively).

To assess the change in K6 distribution over two decades, the distributions of the K6 were first compared between 1997 and 2017 groups (Fig. 2a). Despite the 20-year difference, the two graphs appeared as a single graph because they almost completely overlapped. This finding was in accordance with the observation that the overlap coefficient between 1997 and 2017 groups was 0.97 (Table 2). Generally, a score of $\underline{\underline{ > }}$13 and a score between 5 and 12 on K6 are defined as the cut-point for severe and moderate psychological distress, respectively^3^. The two graphs almost overlapped beyond the two cut-points.

To assess the K6 total score distribution pattern, we evaluated the distributions using a log-normal scale (Fig. 2b). The distributions for 1997 and 2017 exhibited a linear pattern with similar gradients on a log-normal scale, indicating that K6 total scores for the two groups followed an exponential pattern with similar rate parameters on a normal scale. The linear pattern covered a wide range of K6 scores beyond the two cut-points. As indicated by the arrow, at the lower end, the distributions for the two groups exhibited higher frequencies compared to those predicted from the exponential pattern (Fig. 2b).

**Figure 3:**
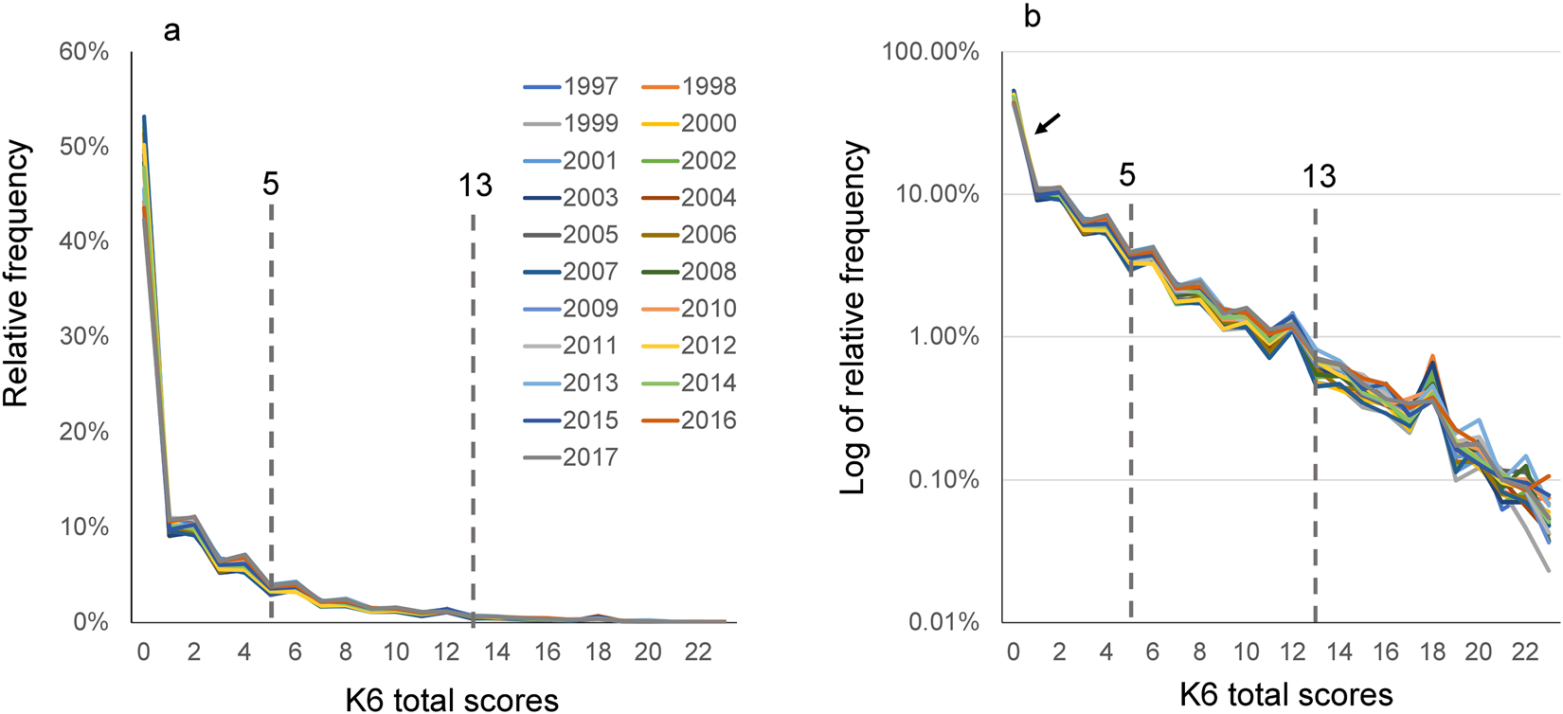
(**a**) Comparison of K6 total score distributions among the 21 different year groups. K6 total score distributions were commonly right-skewed and similar among all year groups. (**b**) On a log-normal scale, the distributions for all year groups exhibited a linear pattern with similar gradients, indicating that K6 total scores for all groups followed an exponential pattern with similar rate parameters. As indicated by arrows, at the lower end, distributions for all groups exhibited higher frequencies compared to those predicted from the exponential pattern. Auxiliary lines indicate the cut-points for moderate and severe psychological distress (a score of 5 and 13, respectively).

The distributions of the K6 total scores were compared among the 21 different year groups (Fig. 3a). The distributions of the K6 total scores were commonly right-skewed and similar among all year groups. It was difficult to distinguish each year group graphically because all 21 graphs were close. These findings were in accordance with the observation that the median of the overlap coefficient was 0.96 (Table 2).

On a log-normal scale, the distributions for all year groups exhibited a linear pattern with similar gradients (Fig. 3b), indicating that K6 total scores for all groups followed an exponential pattern with similar rate parameters. As indicated by the arrow, the distributions for all groups exhibited higher frequencies at the lower end compared to those predicted from the exponential pattern.

Regression curve for an exponential model was calculated from 0–24 points (Supplementary Data, Dataset 1). The rate parameters for all year groups ranged from −0.21 to −0.24, indicating similar gradients across all year groups. The coefficients of determination ranged from 0.91 to 0.96, suggesting that the K6 distributions had good fit to an exponential distribution.

### Graphical analysis of the distributions among different age groups

**Figure 4:**
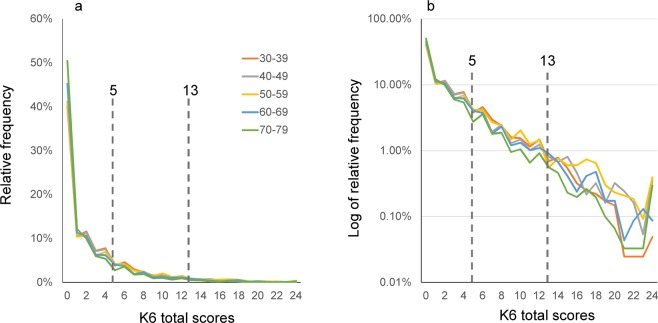
(**a**) Comparison of K6 score distributions between the 30 s, 40 s, 50 s, 60 s, and 70 s groups. K6 score distributions were commonly right-skewed and similar among all age groups. (**b**) On a log-normal scale, the distributions for all year groups exhibited a linear pattern with similar gradients, indicating that K6 total scores for all age groups followed an exponential pattern with similar rate parameters. Compared to the graph including all age participants (Figs 3b and 4b, the curves appeared to fluctuate more randomly as the scores increased, likely reflecting the small sample sizes for higher scores. Auxiliary lines indicate the cut-points for moderate and severe psychological distress (a score of 5 and 13, respectively).

Using 2017 data, the distributions of the K6 total scores were compared among the different age groups during adulthood (30 s, 40 s, 50 s, 60 s, and 70 s) (Fig. 4a). The number for each age group was 4070, 3718, 4334, 4624, and 3048 for the 30 s, 40 s, 50 s, 60 s, and 70 s, respectively. The distributions of K6 total scores were similar among all age groups, suggesting that the distribution of psychological distress was stable between the 30 s and 70 s.

On a log-normal scale, the distributions for different age groups exhibited a linear pattern, indicating that the K6 distributions followed an exponential pattern across different age groups (Fig. 4b). However, compared to the graph including participants of all ages (Figs 2b and 3b), the curves for each age group appeared to fluctuate more randomly as the scores increased (Fig. 4b), likely reflecting the small sample sizes due to age-based classification.

## Discussion

Our study investigated whether the distribution of psychological distress in the US general population was stable in recent decades. Coefficients of variation and linear regression analysis revealed that the mean K6 total scores were stable from 1997 to 2017. Furthermore, overlap coefficients and graphical analysis demonstrated that the distributions of K6 total scores were considerably stable during this period. These findings suggest that apparent changes in the prevalence of psychological distress have not been observed in recent decades due to the stability of psychological distress distribution in recent decades. Furthermore, the distribution has followed an exponential pattern with the exception of the lower end of the score range.

The stable distribution of K6 total scores over two decades may be regarded as a unique feature of psychological distress. As noted in the Introduction, a disease tends to exhibit an apparent upward or downward trend over time. In contrast, the present study demonstrated that the distribution of K6 in the general population has been stable over two decades. In addition, the distribution of psychological distress and depressive symptoms is stable against age^25,26^.

Although a person’s psychological distress can change depending on stressful life events, and may lead to depression and anxiety disorders, the distribution of psychological distress remains stable in the general population. Thus, we hypothesised that there may be a mechanism that stabilises the distribution of psychological distress in the general population. In the Introduction, we highlighted several explanations for the lack of improvement in the prevalence of psychological distress in recent decades. Our assumption differs from those explanations in that the distribution of psychological distress originally had the feature of stability.

Of note, the population-based study conducted in Taiwan using the CHQ demonstrated that the prevalence of common mental disorders increased in parallel with national rates of unemployment and divorce between 1990 and 2010^14^. Although the reason for the inconsistent findings regarding recent time trends of psychological distress is unclear, one possibility is that difference of socioeconomic environment in recent decades may contribute to discrepancies between studies. Indeed, the employment rate in Taiwan increased continuously from the early 1990s to 2010^14^. Another possibility is the differences in the measurement of psychological distress between K6 and CHQ. For example, whereas the K6 is scored with a five-point Likert method (0-1-2-3-4), the study conducted in Taiwan used CHQ which is scored with a binary method (0-0-1-1)^14^. A previous study suggested that the change in depressive symptom scores using the binary method (0-1-1-1) is different from that using the Likert method (0-1-2-3)^39^.

Our results demonstrated that the distribution of K6 total scores follows an exponential distribution with the exception of the lower end. This finding is consistent with previous studies using the Center for Epidemiologic Studies Depression Scale (CES-D), PHQ-9, and Clinical Interview Schedule-Revised (CIS-R)^29–31,40^. It is noteworthy that the exponential pattern covered a wide range of K6 scores beyond the cut-off points for severe and moderate psychological distress. On the other hand, our results indicate that the total K6 score exhibited a non-exponential pattern at the lower end of the distribution (Figs 2b, 3b and 4b). This finding is consistent with results of other studies using the CES-D, PHQ-9, and CIS-R^29,30,40^ Previous research suggests that the response rate of the negative adverb option (e.g., “none of the time”) contributes to the non-exponential pattern at the lower end of the distribution^28,40^. Specifically, the total scores with a high probability for the negative adverb option exhibited higher scores at the lower end of the distribution than those predicted from the exponential pattern. In contrast, the total scores with a low probability for the negative adverb option exhibited lower scores. Indeed, according to our calculations, the average probabilities of “none of the time” for 1997 and 2017 (74.1% and 74.0%, respectively) were higher than that of the National Survey of Midlife Development in the United States data (61.8%), which exhibited an exponential pattern for the whole distribution^41^. Our recent simulation using an ordinal scale model revealed that when the severity of psychological distress followed an exponential distribution, the total scores of a psychological distress scale exhibited an exponential pattern, except at the lower end of the distribution^42^.

Investigating mathematical patterns in observed data is greatly important in the natural sciences. Indeed, a physical phenomenon is usually expressed using a mathematical model. To date, physicists have measured physical phenomena, identified a mathematical pattern in the observed data, and constructed a mathematical model. However, in the field of psychiatry, methodologies for identifying mathematical patterns in empirical data remain underdeveloped. Psychiatry research rather analyses observed data using statistical models, which mostly assume a normal distribution^43^. If the observed distributions of psychological distress are proven to follow a specific non-normal distribution, these statistical models assuming normality (e.g., factor analysis and multiple linear regression analysis) will require reconsideration. Moreover, if the mathematical patterns of psychological distress in a general population are established, they will be useful for predicting the frequency of individuals with a certain score in a population.

The observed exponential pattern of the distribution appears to correspond to the stability of the distribution. Generally, an exponential distribution and a normal distribution differ in their mechanisms of formation. A normal distribution occurs when independent normally distributed random variables are summed^44^. Thus, the summed normal distribution changes its parameters according to changes in the distribution of each variable. In contrast, an exponential distribution occurs when the conditions of exchange among individuals and the stability of the total amount are both satisfied^33^.

In the context of psychological distress, there are several possibilities for these two conditions. With regards to exchange among individuals, people may indirectly and unconsciously contribute to psychological distress of others. For example, when someone is accepted into a prestigious university, this precludes someone else’s chances of also being accepted. People also often evaluate their circumstances from social comparisons^45,46^. Competition and social comparison may contribute to the indirect exchange of psychological distress among individuals. Of note, while these explanations may explain the exchange of mild psychological distress, it seems to be difficult to apply them for mental disorders or severe psychological distress. It remains unclear why the exponential pattern covers a wide range of K6 scores beyond the cut-off points for severe and moderate psychological distress. Regarding the total amount of psychological distress, present and previous research has provided evidence that the total amount of psychological distress (i.e., the average of psychological scores) has remained stable in recent decades^6,8,10^. Further research is necessary to clarify the reasons underpinning the exponential distribution of psychological distress and depressive symptoms.

This analysis has several limitations. We analysed the 1997 to 2017 National Health Interview Survey (NHIS) data and demonstrated that the distribution of psychological distress is stable. During this period, US experienced a global economic crisis, major disasters such as Hurricane Katrina, and an epidemic of terrorism (e.g., September 11 attacks), which substantially impact psychological distress in the community^47–49^. However, the most serious level of social stress in US history, such as the American Civil War, Great Depression, and World War II did not occur during the period of analysis. Further research using data from other countries or other periods is necessary to generalise the findings of this study. Second, we assessed the similarities among distributions using overlap coefficients and graphical analysis. However, one disadvantage of these analyses is the lack of unified descriptors for the interpretation of results. Thus, even after obtaining the results from these procedures, we were unable to describe the extent of similarity using unified descriptors, such as “significantly,” “slightly,” “well,” etc.^50^ Further research is necessary to establish unified descriptors for the interpretation of these analyses. Third, the response rate of the survey and demographic factors were somewhat different among the year groups. Although the distributions of the K6 total scores were considerably stable from 1997 to 2017, the response rate and these factors could have potentially impacted the distribution of psychological distress to a certain extent. Further research is needed to clarify this issue. Finally, our findings should be interpreted with caution due to uncertainties around the diagnosis of mental disorders and use of mental health treatments. In the present study, participants did not receive a standard psychiatric diagnosis. Thus, the results did not encompass a diagnosis of common mental disorders. Furthermore, although the present study demonstrates a stable tendency of the distribution of psychological distress in recent decades, the tendency does not imply that mental health service does not change the prevalence of mental disorders. As noted in the Introduction, much of the increased provision of treatment in recent decades does not meet the needs of individuals in greatest need^6,8^. To evaluate the effects of mental health service on society, it is first necessary to narrow treatment gaps in the general population.

In conclusion, our findings suggest that distribution of the K6 total score has remained stable over the past two decades in the US and follows an exponential pattern with the exception of the lower end. The stability of the K6 distribution may be linked to the exponential distribution. Furthermore, our findings provide a potential explanation for the lack of apparent changes in the prevalence of common mental disorders during the analysed period. Further studies are required to verify whether our findings can be generalised to other countries and/or time periods.

## Methods

### Dataset

We used data from the 1997–2017 NHIS. The NHIS is a national survey designed to monitor the health of the United States^51,52^. NHIS is an annual, cross-sectional survey of non-institutionalized US citizens conducted by the National Center for Health Statistics. NHIS data were collected through personal household interviews. For each participating family, a randomly selected adult aged 18 years or older was invited to participate in the interview. The final response rates, which were calculated by multiplying the response rates for a family interview and random adult interview, ranged from 53% to 80%, with an average of 67%. A total of 646,279 individuals aged 18 years or older participated in the NHIS 1997 to 2017 and comprised the sample for this study.

The present study used de-identified data available to the public. The author’s institutional review board does not consider the secondary analysis of publicly available data as research on human subjects. The data used in this study are available in the NHIS repository, https://www.cdc.gov/nchs/nhis/data-questionnaires-documentation.htm.

### Measures

In the 1997-2017 NHIS, psychological distress was assessed using K6, a standardized screening measure of psychological distress^2^. K6 includes six items related to depressive and anxiety symptomatology and assesses nonspecific psychological distress over the past 30 days. Items are rated on a 5-point Likert scale from “none of the time” (=0) to “all the time” (=4). Total scores range from 0–24.

### Analysis

As we analysed the distribution of K6 scores, participants who did not respond to all K6 items (13,907 individuals: 2.2%) were excluded from this analysis. The final sample for this study consisted of 632,372 individuals. Descriptive statistics such as mean, standard deviation, skewness, kurtosis, and frequency curve were calculated for each year group. We defined a score of ≥13 on K6 as indicative of severe psychological distress. To evaluate the degree of stability in mean K6 total score, we calculated a stability measure, the coefficient of variation for the mean score^53^. To analyse whether psychological distress changed over time, we examined trends in average K6 score by year using a linear regression model. As our hypothesis depended on distribution similarity rather than equality, we did not determine significant differences based on the null hypothesis that the two mean or median values were equal.

**Figure 5:**
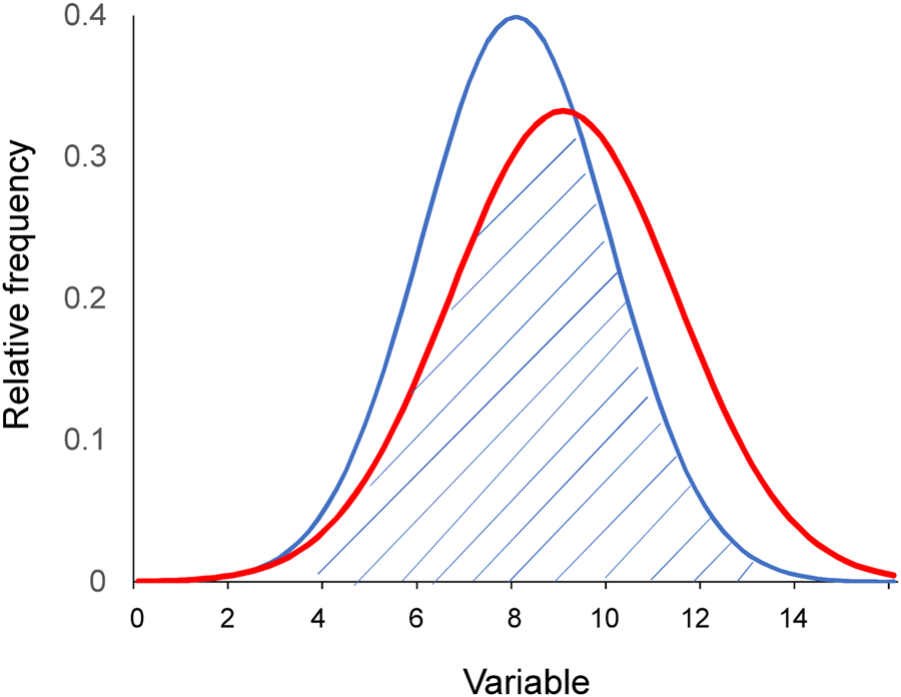
The overlap coefficient from two normal distributions with unequal average and variance (shaded area). 10.3389/fpsyt.2018.00390.

Next, to evaluate the similarity of the distributions between different year groups, we calculated the overlap coefficient of the distributions^54^. Overlap coefficient is a similarity measure that calculates the overlap between two probability distributions (Fig. 5)^54^. Effect sizes such as Cohen’s *d* are often used to quantify the degree of difference between two distributions^55^. However, effect sizes only target the difference between mean or median values and not the pattern or the distribution itself. Thus, we calculated overlap coefficients to measure the similarities among distributions. Overlap coefficients ranged between zero and one.

We used graphical analysis to illustrate the similarity of the distribution patterns among different year groups. We employed both normal and log-normal scales. Log-normal scales enables the identification of the range of an exponential pattern. Regression curves for an exponential model were estimated for each year group.

To confirm the stability of the distribution of psychological distress against age, we investigated whether the distribution of psychological distress remained stable during adulthood in the general population. We used JMP Version 11 for Windows (SAS Institute, Inc., Cary, NC, USA) for descriptive statistics and statistical procedures.

### Ethics and consent to participate

The present study used de-identified data available to the public. The author’s institutional review board does not consider the secondary analysis of publicly available data as research on human subjects.

## Supplementary information

Data set 1
